# Association of obstructive sleep apnea symptoms with all‐cause mortality and cause‐specific mortality in adults with or without diabetes: A cohort study based on the NHANES

**DOI:** 10.1111/1753-0407.13538

**Published:** 2024-04-10

**Authors:** Qian Zhang, Qi Zhang, Xiaomin Li, Gang Du, Xiaojin Feng, Runtao Ding, Yuhua Chi, Yongping Liu

**Affiliations:** ^1^ Department of General Practice Affiliated Hospital of Weifang Medical University Weifang China; ^2^ Department of Endocrinology and Metabolism Affiliated Hospital of Weifang Medical University Weifang China; ^3^ Department of Clinical Research Center Affiliated Hospital of Weifang Medical University Weifang China; ^4^ Judicial appraisal center Affiliated Hospital of Weifang Medical University Weifang China

**Keywords:** diabetes, mortality, NHANES, obstructive sleep apnea syndrome

## Abstract

**Background:**

The association between obstructive sleep apnea syndrome (OSAS) and mortality has not been extensively researched among individuals with varying diabetic status. This study aimed to compare the relationship of OSAS with all‐cause and cause‐specific mortality in US individuals with or without diabetes based on data from the National Health and Nutrition Examination Survey (NHANES).

**Methods:**

The study included participants from the NHANES 2005–2008 and 2015–2018 cycles with follow‐up information. OSAS data (OSAS.MAP10) was estimated from the questionnaire. Hazard ratios (HRs) and the 95% confidence interval (CI) of OSAS for mortality were calculated by Cox regression analysis in populations with different diabetes status. The relationships between OSAS and mortality risk were examined using survival curves and restricted cubic spline curves.

**Results:**

A total of 13 761 participants with 7.68 ± 0.042 follow‐up years were included. In the nondiabetic group, OSAS.MAP10 was positively associated with all‐cause, cardiovascular, and cancer mortality. In individuals with prediabetes, OSAS.MAP10 was positively related to all‐cause mortality (HR 1.11 [95% CI: 1.03–1.20]) and cardiovascular mortality (HR 1.17 [95% CI: 1.03–1.33]). The relationship between OSAS.MAP10 and the risk of all‐cause mortality and cancer mortality exhibited L‐shaped curves in diabetes patients (both with nonlinear *p* values <.01). Further threshold effect analysis revealed that OSAS was positively related to death risk when OSAS.MAP10 exceeded the threshold scores.

**Conclusion:**

The relationship between OSAS and mortality differed among participants with or without diabetes. Individualized clinical treatment plans should be developed in clinical practice to reduce the risk of death for patients with different metabolic conditions.

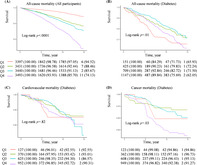

## Graphical abstract

Highlights

- The study examined the association between obstructive sleep apnea symptoms (OSAS) and all‐cause as well as cause‐specific mortality in US individuals with and without diabetes, utilizing data from the National Health and Nutrition Examination Survey.
- Among patients without diabetes, OSAS was positively associated with all‐cause, cardiovascular, and cancer mortality.
- For diabetes patients, the relationship between OSAS and the risk of all‐cause mortality and cancer mortality exhibited L‐shaped curves.

## 1 INTRODUCTION

Obstructive sleep apnea syndrome (OSAS) is characterized by recurring episodes of the upper airway fully or partially collapsing during sleep, resulting in reduced (hypopnea) or absent (apnea) airflow lasting at least 10 s and associated with either cortical arousal or a drop in blood oxygen levels.^1^ Approximately 25% of individuals in the United States suffer from OSAS, affecting 34% of men and 17% of women. It significantly contributes to excessive drowsiness, diminishes quality of life, impairs productivity, and increases the risk of car accidents.^2^, ^3^ Recent studies indicated that severe OSAS increases the risk of cardiovascular and all‐cause death.^4^, ^5^ The risk of cardiovascular and metabolic disease may increase 2–3 times in adults with OSAS.^1^ However, only about 1/50 of people with OSAS symptoms received a diagnosis and treatment.^6^ Nevertheless, current studies mainly focus on general adults, and data on the relationships between OSAS and death in patients with different metabolic states of diabetes are limited.

Type 2 diabetes mellitus (T2DM) has become a global public health concern. According to the International Diabetes Federation, 374 million and 463 million people worldwide had prediabetes and diabetes, respectively, in 2019.^7^ Previous studies have shown that diabetes and prediabetes increase the risk of death from all causes, cardiovascular disease, and cancer,^8^, ^9^ reducing expected lifespan by 4–10 years for individuals aged 40–60 years and independently increasing the risk of death from cardiovascular disease and cancer by 1.3–3.0 times.^10^ Despite substantial evidence linking OSAS to higher all‐cause and cardiovascular mortality,^11^ data on the association between OSAS and mortality among adults with different status of diabetes remain scare.

In this study, we examined the associations between OSAS and mortality in adults with different states of diabetes in the United States using the latest information from the National Health and Nutrition Examination Survey (NHANES),^12^ aiming to establish a theoretical basis for the clinical assessment of the risk of death in adults with different glucose metabolism statuses.

## 2 METHODS

### 2.1 Study population

**FIGURE 1:**
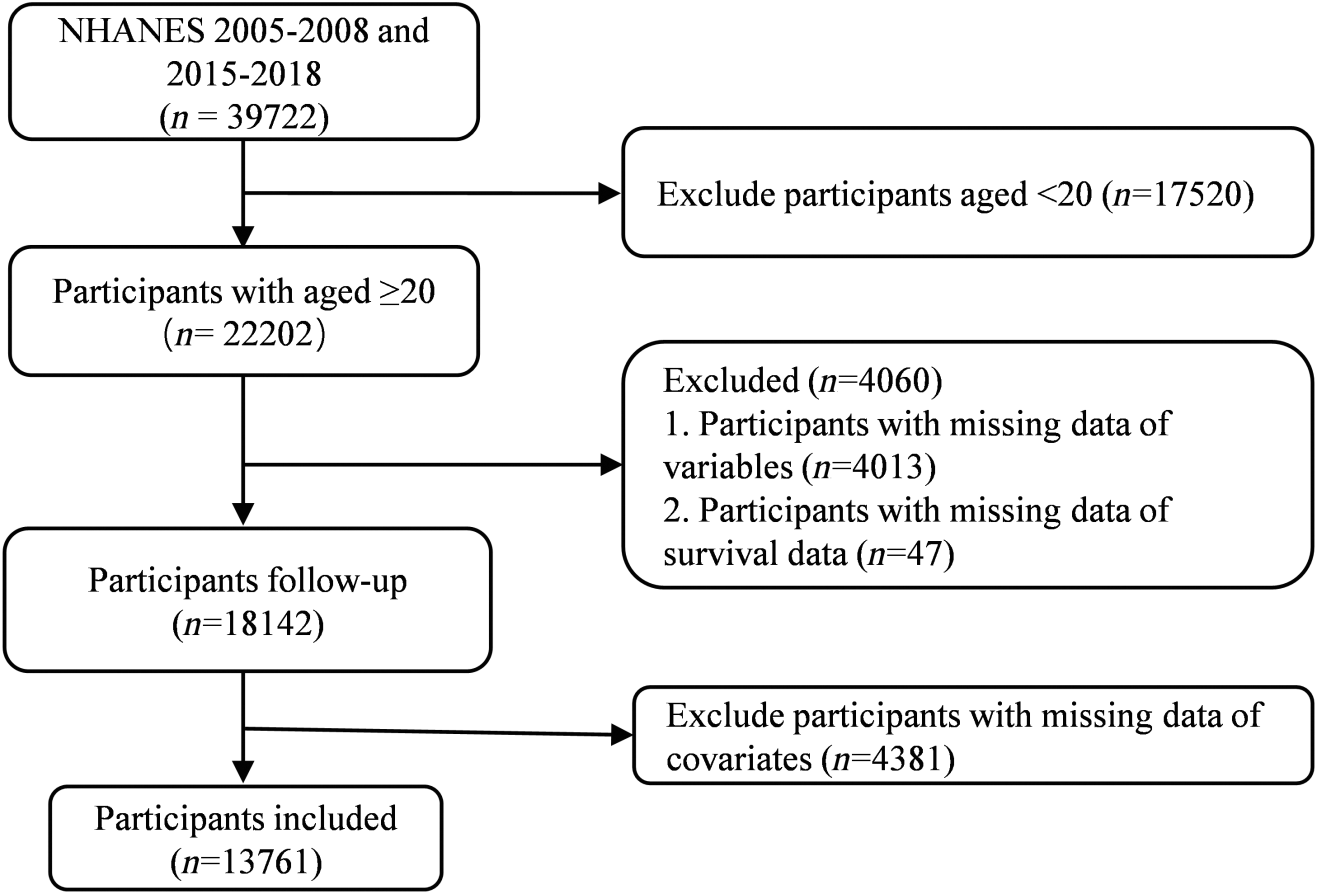
Flow chart of participants in this study. NHANES, National Health and Nutrition Examination Survey.

The NHANES is a longitudinal study with an advanced stratified, multi‐stage, probability cluster sampling design sponsored by the National Centers for Health Statistics to assess the health and nutritional status of the US population. In this study, we collected data from 13 761 participants over 20 years old, including follow‐up and relevant variables, from the NHANES survey cycles of 2005–2008 and 2015–2018 (Figure 1).

### 2.2 Diagnosis of OSAS and the multivariable apnea prediction index

The OSAS was defined based on one of three symptoms: (a) loud snoring more than 3 nights/week; (b) breath retention, sneezing, or wheezing more than 3 nights/week; and (c) feeling overly sleepy during the day 16–30 times/month despite sleeping around 7 or more hours per night. A multivariable apnea prediction (MAP) index (0–1.0) was used to evaluate the risk of OSAS, according to two self‐reported NHANES items of “snoring” and “snort or stop breathing.”.^13^ These items were scored 0, 2, 3, and 4, based on the frequency (never, 1–2 nights/week, 3–4 nights/week, and 5 or more nights/week, respectively). The formula for the MAP index is: MAP index = e^x^/(1 + e^x^), where *x* = −8.160 + 1.299 × Index 1 + 0.163 × body mass index (BMI) − 0.028 × Index 1 × BMI +0.032 × Age + 1.278 × Sex, and where sex = 1 if male and 0 if female, and Index 1 is the mean score of the two self‐reported items.^14^ For better explanation in this study, multiply the MAP index value by 10 and define it as OSAS.MAP10.

### 2.3 Definition of different diabetes statuses

Diabetes was diagnosed based on one of the following conditions: (a) medical diagnosis by a health care professional; (b) hemoglobin A1c (HbA1c) >6.5%; (c) fasting blood glucose (FBG) >7.0 mmol/L; (d) a random or 2‐h oral glucose tolerance test (OGTT) blood sugar >11.1 mmol/L; or (e) taking of diabetes medication or insulin. One of the following criteria was sufficient for a diagnosis of prediabetes: (a) doctor informed prediabetes; (b) HbA1c between 5.7% and 6.5%; (c) FBG in the range of 5.6 and 7.0 mmol/L; or (d) OGTT 2‐h blood sugar between 7.8 mmol/L and 11.1 mmol/L. Other condition was deemed to have normal glucose levels. HbA1c was measured at baseline. Diabetes medication use was classified as none, oral medication or insulin, or other based on self‐reported data.

### 2.4 Other variable definition

Participants self‐reported their age, sex, race or ethnicity, education, smoking, and drinking status. Race/ethnicity categories included Hispanic, non‐Hispanic Black, non‐Hispanic White, and others. The BMI (kg/m^2^) was calculated using the formula weight divided by height squared. Family poverty income ratio was classified as 0–1.0, 1.0–3.0, or >3.0.^15^ Education was divided into three categories: college and above, high school or equivalent, and less than high school. Smoking status was divided into three categories as follows: never (<100 cigarettes in a lifetime), former (>100 cigarettes in a lifetime but no longer smoke), and current (>100 cigarettes in a lifetime and smoke at times or daily).^16^ Drinking status was categorized as (a) never drinking, (b) former drinking (having drunk before and not drinking in the last 12 months), (c) mild alcohol user (<2 drinks per day for females, <3 drinks per day for males, or excessive drinking <2 days per month), (d) current moderate drinking (≥2 drinks per day for females, ≥3 drinks per day for males, or excessive drinking ≥2 days per month), and (e) current heavy drinking (≥3 drinks per day for females, ≥4 drinks per day for males, or excessive drinking more than 5 days per month).^17^

Asthma and chronic obstructive pulmonary disease (COPD) were diagnosed through a combination of patient self‐report, medical history, medication use, and age. Chronic kidney disease was defined by a urine albumin‐creatinine ratio of less than 30 mg/g or an estimated glomerular filtration rate below 60 mL/min/1.73 m^2^.^18^ Hypertension was defined as meeting one or more of the following criteria: (a) using hypertensive medication, (b) being diagnosed with hypertension by a physician, (c) having a systolic blood pressure of greater than 140 mm Hg or a diastolic blood pressure higher than 90 mm Hg on three separate occasions. Hyperlipidemia can be diagnosed if any of the following conditions were met: (a) high triglycerides (≥150 mg/dL), (b) high cholesterol (≥200 mg/dL or low‐density lipoprotein cholesterol ≥130 mg/dL or high‐density lipoprotein cholesterol ≤40 mg/dL (male), 50 mg/dL (female), or (c) use of lipid‐lowering medications. Cardiovascular disease was identified by self‐reported presence of coronary heart disease, congestive heart failure, heart attacks, strokes, and angina pectoris. The NHANES Laboratory/Medical Technologists Procedures Manual provided details about the strict procedures for blood collection and analysis.

### 2.5 All‐cause and cause‐specific mortality

Mortality data was obtained from the publicly‐accessible National Death Index recorded until December 31, 2019 (https://www.cdc.gov/nchs/datalinkage/mortality-public.htm).^19^ The follow‐up period was calculated by subtracting the date of the baseline examination from the participant's final reported living or censoring date in the mortality file. The study focused on all‐cause mortality, cardiovascular mortality, and cancer mortality, as determined by the *International Classification of Diseases, Tenth Revision* (ICD‐10). Cancer mortality was categorized as C00‐C97, and cardiovascular mortality was defined by ICD‐10 codes I00‐I09, I11, I13, or I20‐I51.

### 2.6 Statistical analysis

Given the design characteristics of NHANES, we selected suitable sample weight applied for all the statistical analyses. The independent‐samples weighted *t*‐test was performed to contrast the two categories. Data from the enumeration were given as the number and the composition ratio (*n* [%]), and the difference between groups was weighted with a one‐way analysis of variance or *χ* ^2^‐test. First, we compared demographic characteristics among participants with different diabetes statues. Univariate difference analysis yielded potential confounders. Three Cox proportional hazards models adjusted different covariates, shown as adjusted for hazard ratio (HR) with 95% confidence interval (95% CI), were created to explore the connections of OSAS risk with all‐cause and specific mortality in adults with different status of diabetes. We performed Kaplan–Meier survival curves to analyze the relationship between OSAS and mortality in participants with different metabolic states of diabetes. Restricted cubic spline (RCS) curves were performed to examine the nonlinear relationship between OSAS risk scores and all‐cause and specific mortality in different diabetic statuses.

We also performed sensitivity studies to ensure the robustness of our findings. First, individuals who passed away within 2 years of baseline were removed to prevent reverse causation. Second, we eliminated diabetics who were taking drugs that interfere with metabolism to rule out the effects of the drugs. Third, individuals with cardiovascular disease at baseline were excluded. All the statistical analysis were performed using R (4.2.2). A *p* < .05 was considered statistically significant.

## 3 RESULTS

### 3.1 Demographic features of US adults based on diabetes status

This study included 18 142 people, with a median OSAS.MAP10 score of 3.87 ± 0.04 (mean ± SE). Of them, there were 3283 diabetes with a score of 5.68 ± 0.05, and 5218 prediabetes with a score of 4.63 ± 0.05. Adults with diabetes were older, had a higher BMI, were more probable to be male, and had a greater level of education than people without diabetes (Table 1). Diabetic individuals were more likely to be never or former smoker, to drink in moderation, and to have a greater prevalence of hypertension, hyperlipidemia, asthma, COPD, chronic kidney disease, and heart disease (Table 1). The proportions of diabetes and prediabetes were much higher in population with OSAS than without (*p* < .001) (Table S1). Based on OSAS.MAP10 quartiles, all the participants were divided into Q1, Q2, Q3, and Q4. Among those with diabetes, participants with higher OSAS.MAP10 were more probable to be older men, non‐Hispanic White, obese, and less inclined to be heavy drinkers and smokers now (Table S2).

**TABLE 1:** Demographic characteristics of US adults with different diabetic status.

Characteristic	*N*	Overall, *N* = 18 142 (100%)	Diabetes status			*p* value^b^
			Non‐diabetes, *N* = 9641 (58%)^a^	Pre‐diabetes, *N* = 5218 (28%)^a^	Diabetes, *N* = 3283 (14%)^a^	
Age, years	18 142	46.7 (0.3)	41.6 (0.3)	51.4 (0.3)	58.9 (0.4)	**<.001**
Sex	18 142					**<.001**
Female		9238 (51%)	5264 (54%)	2431 (46%)	1543 (49%)	
Male		8904 (49%)	4377 (46%)	2787 (54%)	1740 (51%)	
Race or ethnicity	18 142					**<.001**
Hispanic		1752 (5.5%)	870 (5.4%)	532 (5.6%)	350 (5.8%)	
Non‐Hispanic Black		3925 (11%)	1962 (10%)	1128 (11%)	835 (14%)	
Non‐Hispanic White		7392 (67%)	4222 (69%)	2032 (65%)	1138 (62%)	
Other		5073 (16%)	2587 (15%)	1526 (18%)	960 (18%)	
BMI, kg/m^2^	18 142					**<.001**
<25		5169 (30%)	3528 (39%)	1172 (21%)	469 (12%)	
25–30		6044 (32%)	3246 (32%)	1845 (36%)	953 (26%)	
≥30		6929 (37%)	2867 (29%)	2201 (43%)	1861 (61%)	
Education	18 130					**<.001**
College and above		9415 (61%)	5326 (63%)	2681 (59%)	1408 (52%)	
High school or equivalent		4256 (24%)	2258 (24%)	1215 (25%)	783 (27%)	
Less than high school		4459 (15%)	2053 (13%)	1317 (16%)	1089 (21%)	
Family income poverty ratio	16 538					**<.001**
≤1		3237 (13%)	1681 (13%)	904 (12%)	652 (15%)	
1–3		7078 (36%)	3662 (35%)	2044 (35%)	1372 (41%)	
>3		6223 (52%)	3492 (53%)	1805 (52%)	926 (44%)	
Smoking status	18 131					**<.001**
Former		4323 (24%)	1855 (20%)	1393 (28%)	1075 (33%)	
Never		10 159 (55%)	5702 (58%)	2768 (52%)	1689 (51%)	
Now		3649 (20%)	2078 (21%)	1052 (19%)	519 (16%)	
Drinking status	15 824					**<.001**
Never		2318 (11%)	1160 (10%)	641 (9.9%)	517 (16%)	
Former		2357 (12%)	1019 (9.8%)	673 (12%)	665 (19%)	
Mild		5392 (38%)	2748 (35%)	1715 (43%)	929 (38%)	
Moderate		2557 (18%)	1556 (21%)	673 (16%)	328 (14%)	
Heavy		3200 (22%)	1990 (25%)	846 (19%)	364 (13%)	
HbA1c, %	17 228	5.59 (0.01)	5.23 (0.01)	5.60 (0.01)	7.04 (0.04)	**<.001**
OSAS.MAP10	18 142	3.87 (0.04)	3.08 (0.05)	4.63 (0.05)	5.68 (0.05)	**<.001**
COPD	18 141	774 (3.7%)	257 (2.3%)	276 (5.2%)	241 (6.7%)	**<.001**
Asthma	18 142	2524 (14%)	1309 (14%)	698 (14%)	517 (16%)	.3
Chronic kidney disease	17 061	3161 (14%)	995 (9.1%)	894 (14%)	1272 (36%)	**<.001**
Hypertension	18 141	7467 (37%)	2658 (25%)	2479 (45%)	2330 (69%)	**<.001**
Hyperlipidemia	18 140	12 310 (67%)	5555 (58%)	3946 (77%)	2809 (87%)	**<.001**
Cardiovascular disease	18 140	1945 (8.1%)	533 (4.1%)	586 (9.2%)	826 (23%)	**<.001**
Diabetes medication use	18 130					**<.001**
None		7968 (44%)	5414 (53%)	2147 (39%)	407 (11%)	
Other		8065 (48%)	4216 (47%)	3059 (61%)	790 (26%)	
Oral medication or insulin		2097 (8.6%)	6 (<0.1%)	7 (0.1%)	2084 (63%)	

Abbreviations: BMI, body mass index; COPD, chronic obstructive pulmonary disease; HbA1c, hemoglobin A1c; OSAS.MAP10, obstructive sleep apnea symptoms multivariable apnea prediction *10.

^a^ Mean (SD) for continuous variable; *n* (%) for categorical variable.

^b^ Wilcoxon rank‐sum test for complex survey samples; chi‐square test with Rao and Scott's second‐order correction. A *p* < .05 was considered statistically significant (Bold).

### 3.2 Association between OSAS.MAP10 with all‐cause and cause‐specific mortality in the US adults with or without diabetes

We further excluded participants with missing data of covariates, and eventually 13 761 individuals were enrolled in the study. With 7.68 ± 0.042 follow‐up years, 1385 fatalities (of which 345 cardiovascular deaths and 319 cancer deaths) were recorded. As shown in Table 2, the OSAS.MAP10 was positively related to all‐cause, cardiovascular, and cancer mortality in adults without diabetes in model 1, which adjusted for age, sex, race, and BMI (HR 1.20 [95% CI: 1.16–1.24] for all‐cause mortality, HR 1.30 [95% CI: 1.22–1.39] for cardiovascular mortality, and HR 1.23 [95% CI: 1.13–1.34] for cancer mortality, respectively). These relationships did not change in model 2, which adjusted for covariates in model 1 and other death risk factors such as education, smoking status, drinking status, and family poverty income ratio. However, this relationship disappeared for cardiovascular and cancer in model 3, adjusting for the covariates in model 2 and other background diseases including COPD, asthma, cardiovascular disease, hypertension, hypercholesterolemia, and chronic kidney disease.

**TABLE 2:** Weighted association between OSAS.MAP10 with all‐cause mortality and cause‐specific mortality in different status of diabetes.

Group	Characteristic	Nondiabetes			Prediabetes			Diabetes		
		HR	95% CI	*p* value	HR	95% CI	*p* value	HR	95% CI	*p* value
All‐cause mortality	Model 1	1.20	1.16–1.24	**<.001**	1.22	1.16–1.29	**<.001**	1.31	1.24–1.38	**<.001**
	Model 2	1.17	1.13–1.22	**<.001**	1.23	1.14–1.31	**<.001**	1.24	1.17–1.32	**<.001**
	Model 3	1.06	1.01–1.10	**.008**	1.11	1.03–1.20	**.005**	1.15	1.07–1.24	**<.001**
	Model 3a							1.16	1.08–1.25	**<.001**
Cardiovascular mortality	Model 1	1.30	1.22–1.39	**<.001**	1.29	1.18–1.40	**<.001**	1.39	1.29–1.50	**<.001**
	Model 2	1.24	1.16–1.33	**<.001**	1.31	1.17–1.47	**<.001**	1.33	1.22–1.45	**<.001**
	Model 3	1.07	0.97–1.18	.2	1.17	1.03–1.33	**.015**	1.22	1.07,1.39	**.003**
	Model 3a							1.24	1.08–1.41	**.002**
Cancer mortality	Model 1	1.23	1.13–1.34	**<.001**	1.18	1.08–1.29	**<.001**	1.48	1.35–1.62	**<.001**
	Model 2	1.20	1.08–1.34	**.001**	1.17	1.06–1.30	**<.001**	1.38	1.24–1.54	**<.001**
	Model 3	1.11	0.99–1.25	.070	1.06	0.93–1.21	.4	1.37	1.21–1.56	**<.001**
	Model 3a							1.40	1.23–1.60	**<.001**

*Note*: Model 1: adjusted for age, sex, race/ethnicity, and BMI. Model 2: adjusted for all variables in model 1 and other risk factors for death, including education, smoking status, drinking status, and family income poverty ratio. Model 3: adjusted for all variables in model 2 and other risk factors for death, including cardiovascular disease, hypertension, hypercholesterolemia, chronic obstructive pulmonary disease, asthma, and chronic kidney disease. Model 3a: in adults with diabetes, further adjusted for HbA1c and diabetes medication usage. A *p* < .05 was considered statistically significant (Bold).

Abbreviations: 95% CI, 95% confidence interval; BMI, body mass index; HbA1c, hemoglobin A1c; HR, hazard ratios; OSAS.MAP10, obstructive sleep apnea symptoms multivariable apnea prediction *10.

In adults with prediabetes, the OSAS.MAP10 also increased the risk of all‐cause, cardiovascular, and cancer mortality in all three models, except that the relationship for cancer mortality (HR 1.06 [95% CI: 0.93–1.21]) disappeared in model 3.

In adults with diabetes, OSAS.MAP10 increased the risk of all‐cause, cardiovascular, and cancer mortality in adults with diabetes in model 3a, adjusting for covariates in model 3 and diabetes medication usage, with HR 1.16 (95% CI: 1.08–1.25) for all‐cause mortality, HR 1.24 (95% CI: 1.08–1.41) for cardiovascular mortality, and HR 1.40 (95% CI: 1.23–1.60) for cancer mortality, respectively.

We further analyzed the overall and cause‐specific mortality rates according to different diabetes statuses and genders. The results differed when gender was taken into account. In the male population without diabetes, after adjusting for all variables in model 2 and other risk factors for death, including cardiovascular disease, hypertension, hypercholesterolemia, chronic obstructive pulmonary disease, asthma, and chronic kidney disease, OSAS.MAP10 was found to increase the cardiovascular mortality rate, with HR of 1.13 (95% CI: 1.05, 1.20) (Table S3). Among participants with prediabetes, there was no association between OSAS.MAP10 and overall or cause‐specific mortality rates in both males and females after adjusting for all variables (Table S4). For male participants with diabetes, OSAS.MAP10 was not associated with overall or cause‐specific mortality rates in the adjusted model 3a (all *p* values >.05). In female participants with diabetes, OSAS.MAP10 was associated with a decrease in overall mortality after fully adjusting for model 3 (HR 0.88, 95% CI: 0.79, 0.97). This association remained even after further adjusting for HbA1c and diabetes medication usage in Model 3a (Table S5).

### 3.3 OSAS.MAP10 was nonlinearly related to all‐cause mortality and cancer mortality in the US population with diabetes

**FIGURE 2:**
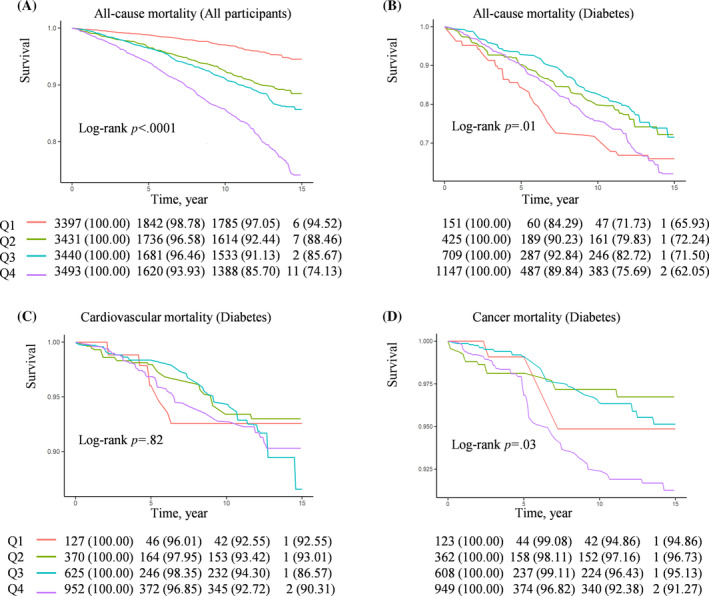
Kaplan–Meier survival curves (crude) were performed to analyze the difference of mortality among the obstructive sleep apnea symptoms multivariable apnea prediction *10 (OSAS.MAP10) quartiles groups (Q1–Q4). (A) Kaplan–Meier survival curves of all‐cause mortality for all participants; (B) Kaplan–Meier survival curves of all‐cause mortality for diabetes; (C) Kaplan–Meier survival curves of cardiovascular mortality for diabetes; (D) Kaplan‐Meier survival curves of cancer mortality for diabetes.

In a quartile Kaplan–Meier analysis based on OSAS.MAP10, Q4 group (Q4, OSAS.MAP10 >6.23) showed a significant reduction in survival time in general participants (Figure 2A). The Q1 groups (Q1, OSAS.MAP10 < 1.70) in both nondiabetic participants and participants with prediabetes shown the lowest rates of all‐cause, cardiovascular, and cancer mortality and the Q4 shown the highest mortality rates (Figure S1A,B). Interestingly, in participants with diabetes, the Q1 and Q4 groups showed a higher risk, but the Q2 and Q3 groups (Q2, OSAS.MAP10 1.70–3.84; Q3, OSAS.MAP10 3.84–6.23) showed lower rates of all‐cause mortality, cardiovascular mortality, and cancer mortality (Figure 2B–D). Therefore, we further performed RCS curves to detect if there was a nonlinear relationship between OSAS.MAP10 with all‐cause and specific mortality in patients with diabetes.

**FIGURE 3:**
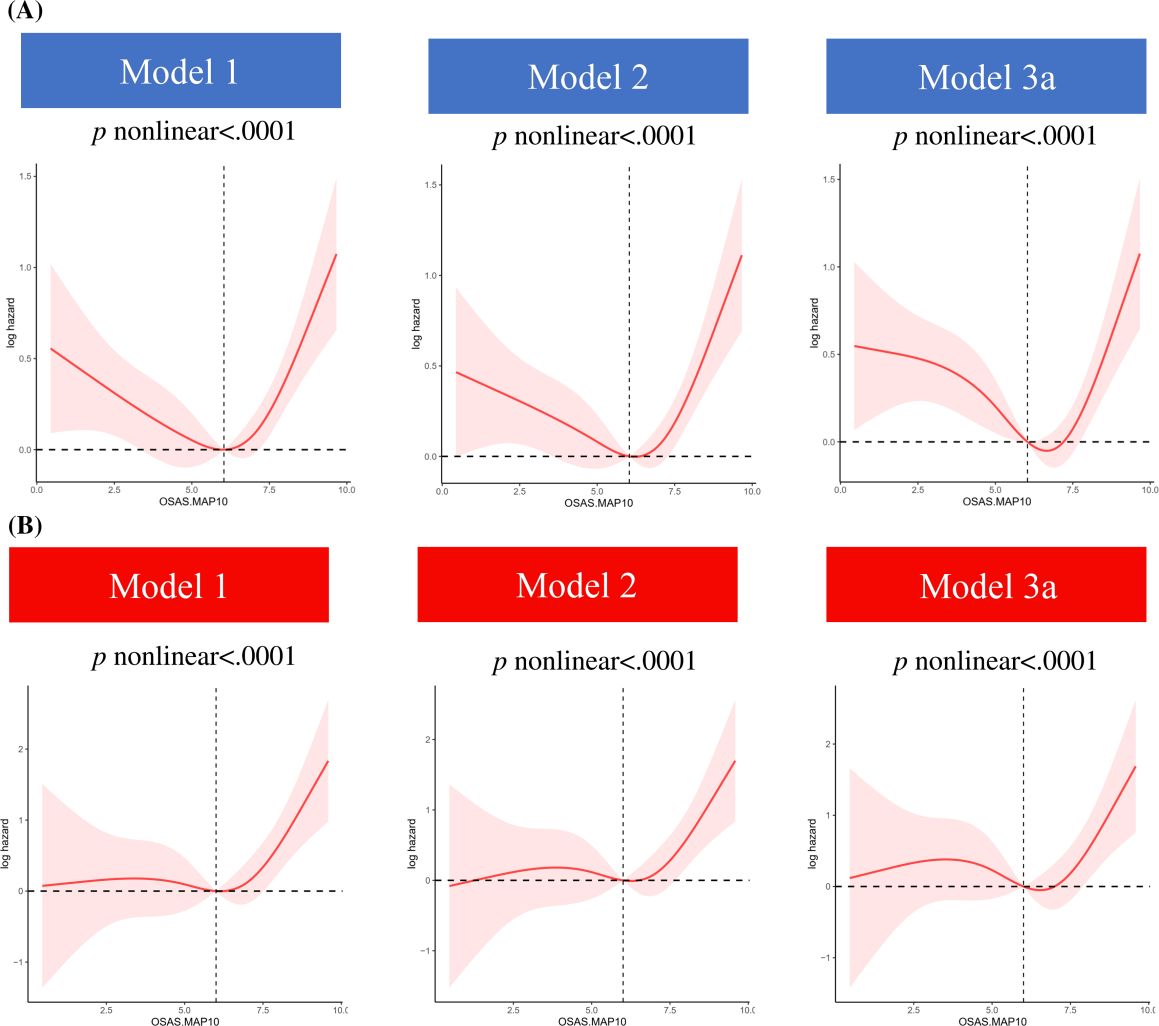
Restricted cubic spline (RCS) curves to detect the nonlinear relationship between obstructive sleep apnea symptoms multivariable apnea prediction *10 (OSAS.MAP10) with all‐cause mortality (A) and cancer mortality (B) in individuals with diabetes.

As Figure 3 depicts, the relationship between OSAS.MAP10 with the risk of all‐cause mortality (Figure 3A) and cancer mortality (Figure 3B) showed L‐shaped curves in patients with diabetes (both nonlinear *p* values <.01). Furthermore, threshold effect analysis was conducted using two‐piecewise Cox regression. We found OSAS.MAP10 significantly increased the risk of all‐cause mortality (HR 1.42 [95% CI: 1.11–1.82] in model 3a, Table 3) and cancer mortality (HR 1.77 [95% CI: 1.02–3.04] in model 2, Table 4) when OSAS.MAP10 was higher than threshold scores (6.65 for all‐cause mortality and 6.51 for cancer mortality). The relationship disappeared when OSAS.MAP10 was below the threshold scores.

**TABLE 3:** Association of OSAS.MAP10 with all‐cause mortality in diabetic participants.

		OSAS.MAP10 < 6.65			OSAS.MAP10 ≥ 6.65		
Group	Characteristic	HR	95% CI	*p* value	HR	95% CI	*p* value
All‐cause mortality	Model 1	0.90	0.81–1.01	.067	1.42	1.10–1.82	**.006**
	Model 2	0.91	0.82–1.02	.10	1.38	1.06–1.81	**.017**
	Model 3a	0.90	0.81–1.01	.075	1.42	1.11–1.82	**.005**

*Note*: Model 1: adjusted for age, sex, race or ethnicity, and BMI. Model 2: adjusted for all variables in model 1 and other risk factors for death, including education, smoking status, drinking status, and family income poverty ratio. Model 3a: adjusted for all variables in model 2 and other risk factors for death, including cardiovascular disease, hypertension, hypercholesterolemia, chronic obstructive pulmonary disease, asthma, chronic kidney disease, HbA1c, and diabetes medication usage. A *p* < .05 was considered statistically significant (Bold).

Abbreviations: 95% CI, 95% confidence intervals; BMI, body mass index; HbA1c, hemoglobin A1c; HR, hazard ratios; OSAS.MAP10, obstructive sleep apnea symptoms multivariable apnea prediction *10.

**TABLE 4:** Association of OSAS.MAP10 with cancer mortality in diabetic participants.

		OSAS.MAP10 < 6.51			OSAS.MAP10 ≥ 6.51		
Group	Characteristic	HR	95% CI	*p* value	HR	95% CI	*p* value
Cancer‐mortality	Model 1	1.00	0.78–1.29	>.9	1.93	1.16–3.21	**.011**
	Model 2	1.04	0.79–1.37	.8	1.77	1.02–3.05	**.041**
	Model 3a	1.04	0.82–1.32	.7	2.01	0.96–4.21	.064

*Note*: Model 1: adjusted for age, sex, race or ethnicity, and BMI. Model 2: adjusted for all variables in model 1 and other risk factors for death, including education, smoking status, drinking status, and family income poverty ratio. Model 3a: adjusted for all variables in model 2 and other risk factors for death, including cardiovascular disease, hypertension, hypercholesterolemia, chronic obstructive pulmonary disease, asthma, chronic kidney disease, HbA1c, and diabetes medication usage. A *p* < .05 was considered statistically significant (Bold).

Abbreviations: 95% CI, 95% confidence intervals; BMI, body mass index; HbA1c, hemoglobin A1c; HR, hazard ratios; OSAS.MAP10, obstructive sleep apnea symptoms multivariable apnea prediction *10.

### 3.4 Stratified analysis

Stratified analyses were performed based on the age, sex, BMI, race or ethnicity, and education level (Table S6). For the participants with diabetes, the associations between OSAS.MAP10 and all‐causes mortality were significantly higher in males, non‐Black participants, and those with education level less than high school. Significant interactions were found between OSAS.MAP10 with sex or BMI (both *p* for interaction were <.05).

### 3.5 Sensitivity tests

We further performed sensitivity analysis to verify the stability of the results. First, we removed individuals who passed away during 2 years from the start of follow‐up (Table S7) or participants with cardiovascular diseases (Table S8), and the results did not change significantly in all‐cause mortality. After excluding patients who were using diabetic medication, the relationship disappeared (Table S9). Interestingly, there was a significant correlation found for cancer mortality, when those with cardiovascular disease and those using diabetic medication were removed, the relationship between OSAS.MAP10 and cancer mortality was strengthened (Tables S10 and S11). However, this relationship was diminished when people who died within the first 2 subsequent years were removed (Table S12).

## 4 DISCUSSION

In our current study, we discovered that OSAS scores were positively linked to overall mortality and specific mortality in nondiabetic and prediabetic groups. However, in diabetic patients, the relationship showed L‐shaped curves. In these patients, the risk of overall mortality and cancer mortality increased by 42% and 77% for each one‐unit increase in OSAS.MAP10 when it exceeded the corresponding threshold values (OSAS.MAP10 ≥ 6.65 for overall mortality and OSAS.MAP10 ≥ 6.51 for cancer mortality, respectively).

It is worth noting that OSAS and diabetes are common comorbidities associated with obesity. A study involving obese diabetic patients found that 86% of them had some degree of OSAS.^20^ Weight loss trials have shown improvements in diabetes treatment and a reduction in the severity of sleep apnea.^21^ Intermittent hypoxia (IH) is a hallmark of OSAS. Experimental studies in animal and cellular models have shown that IH leads to attenuated glucose‐induced insulin secretion in pancreatic β‐cells and enhanced insulin resistance in peripheral tissues and cells.^22^ There are established connections between obesity and insulin resistance. A study involving 40 adults with moderate to severe obstructive sleep apnea revealed that short‐term continuous positive airway pressure treatment improved insulin sensitivity only in the nonobese subgroup.^23^ Therefore, one possible explanation for this L‐shaped curve relationship is the interaction of obese in the correlation between IH and diabetes.

There is a relationship between OSAS severity and cardiovascular disease death risk. A meta‐analysis found that OSAS increased the risk of all‐cause mortality (HR 1.86) and cardiovascular mortality (HR 2.36).^24^ The increased risk of cardiovascular death from OSAS severity is thought to be the result of a combination of causes. On one hand, numerous studies have shown a strong association between OSAS and the occurrence of cardiovascular diseases, such as hypertension, atrial fibrillation, coronary artery disease, congestive heart failure, myocardial infarction, stroke, and overall cardiovascular and disease‐specific mortality rates.^25^ This process is closely linked to increased sympathetic nervous system activity, inflammation, endothelial dysfunction, and elevated blood pressure, all of which contribute to the increased incidence and mortality rates of cardiovascular diseases.^26^, ^27^ On the other hand, there is evidence suggesting that obstructive sleep apnea can lead to the development of cardiovascular diseases, and continuous positive airway pressure therapy can reduce this risk.^28^ Therefore, among the public, close monitoring of OSAS is needed to reduce the risk of cardiovascular diseases and relative mortality.

Current evidence on the association between OSAS severity and cancer development and progression is limited and inconsistent.^29^, ^30^, ^31^ A population‐based study with 22 years follow‐up showed a significant correlation between OSA severity and cancer mortality.^31^ Significant relationships were discovered between OSAS risk markers (hypoxia and sleep fragmentation) and cancer‐specific mortality in a multicenter clinical cohort research involving 2222 participants, even after adjusting for recognized risk factors for cancer development.^32^ One of these studies showed an independent association between the apnea‐hypopnea index and cancer mortality.^31^ A local investigation revealed a substantial relationship between the respiratory disturbance index and cancer mortality.^33^ However, there are also inconsistent result.^34^ Cyclic IH (CIH) is assumed to be the main mechanism for OSA and increased cancer risk.^28^ Animal models have demonstrated that CIH increases cancer progression and that CIH increases cancer risk by accelerating tumor growth,^35^ increasing tumor angiogenesis,^36^ increasing metastasis,^37^ and decreasing immune surveillance.^38^ Therefore, a personalized clinical treatment plan should be made according to individual's situation to reduce the cancer mortality of patients with different metabolic states.

In our research, we observed a higher mortality rate among the diabetic population, which of course can be partially attributed to the epidemiological characteristics of advanced age and multiple comorbidities prevalent in this group. Comorbidities such as hypertension, hyperlipidemia, and heart disease are mostly associated with an increased death risk of diabetes.^39^ In addition, mortality of diabetes can be influenced by behavioral elements such as physical activity, diet, smoking, alcohol consumption, and weight also exert a significant impact. For instance, when infected with SARS‐CoV‐2, diabetic mice with obesity fed with a high‐fat diet have increased mortality compared to control mice.^40^

In this study, meanwhile, we also observed higher OSAS.MAP10 levels in diabetes population. To clarify the relationship between OSAS and mortality in diabetic population clearly, we meticulously controlled for various confounding factors such as age, BMI, gender, smoking, alcohol consumption, and various comorbidities with several models in logistic regression analysis. Even after adjusting for these factors, our study results remained statistically significant.

Studies have revealed that the glycemic control was associated with overall mortality.^41^ To clarify the relationship between OSAS and mortality in diabetic population clearly, we further adjusted the use of diabetes medications and HbA1c. The result revealed increased the overall mortality rate after controlling for these variables in diabetic patients. It has been reported that HbA1c exposure, especially the HbA1c variability, was a risk factor for mortality in patients with diabetes. It can serve as an indicator for glycemic control target in diabetes.^42^ A meta‐analysis comparing the efficacy of antidiabetic drugs in type 2 diabetes found that glucagon‐like peptide‐1 receptor agonists and sodium‐glucose cotransporter‐2 (SGLT‐2) inhibitors have favorable effects on certain cardiovascular outcomes in patients with increased cardiovascular risk.^43^, ^44^ A cohort study found that the use of SGLT‐2 inhibitors can reduce overall mortality in patients with type 2 diabetes.^45^ Therefore, for those patients undergoing the latest therapeutic approaches, effects on mortality from antidiabetic medications need to be taken into account.

Interestingly, we found that OSAS.MAP10 is a protective factor for all‐cause mortality in women with diabetes in our study. However, no specific mechanism has been proposed yet. One possible mechanism is that as the risk of OSA increases, women with diabetes may become more health conscious and adopt healthier behaviors, such as improving their diet and increasing exercise. Additionally, women with diabetes may be more compliant with medication treatment to control their diabetes and related health issues, which may also help to reduce all‐cause mortality. These factors may work together to decrease all‐cause mortality. The relationship between OSAS with mortality in female diabetic patients need to be confirmed by further large‐scale studies.

## 5 STRENGTHS AND LIMITATIONS

This study's strengths included its prospective research methodology, relatively large sample size, and utilization of a nationally representative sample of US citizens with diabetes, which facilitated the applicability of the results. Furthermore, because of the extensive data available in NHANES, we were able to take a variety of possible confounding variables into account, including socioeconomic level, race and ethnicity, lifestyle variables, and comorbidity. However, several limitations should also be considered. First, although the three symptoms mentioned previously were used to define OSAS, the current study investigated relevant information only through questionnaires, which may reduce the degree of association. There is a lack of sleep studies to further identify OSAS. Second, despite correcting for age, gender, ethnicity, BMI, and comorbidities in the model, bias exists as diabetes and OSAS have similar epidemiological characteristics. Third, although the results stayed significant after additional adjustments for diabetes medication usage, HbA1c levels, and the amount of self‐reported multiple disorders, the current study lacked precise information on diabetes. Fourth, information was obtained mainly through questionnaires, which may have information recall bias. Fifth, linkage to national mortality indices was used to determine mortality outcomes, which may lead to misclassification, although previous validation studies showed the method to be highly accurate. Sixth, the current study cannot rule out the influence of psychological stress, genetic predisposition, lingering or unidentified confounding variables, or chance.

## 6 CONCLUSION

In conclusion, our research revealed that mortality and OSAS had varied associations in patients with or without diabetes. The OSAS was positively associated with mortality in the nondiabetic and prediabetic populations. However, in the diabetic individuals, the relationship between OSAS with all‐cause mortality and cancer mortality both shown L‐shaped curves. In these patients, OSAS may significantly increase mortality when it was greater than the threshold. Therefore, an individual clinical treatment plan should be formulated in clinical practice to reduce the danger of death for patients with different metabolic conditions.

## AUTHOR CONTRIBUTIONS

The idea and conceptual design: Yongping Liu and Yuhua Chi. Data acquisition: Qian Zhang and Xiaomin Li. Literature retrieval and data collation: Qian Zhang, Qi Zhang, and Gang Du. Data analysis: Qi Zhang and Xiaojin Feng. Visualization: Qian Zhang and Runtao Ding. Preparation of the original draft: Qian Zhang and Yongping Liu. Manuscript editing: Qian Zhang, Yuhua Chi, and Yongping Liu.

## DISCLOSURE

The authors declare no competing interests.

## CONSENT FOR PUBLICATION

All authors have read and agreed to publish this version of the manuscript.

## Supporting information


**Data S1.** Supplementary Information.

## Data Availability

The datasets generated and/or analyzed during the current study are available in the NHANES (https://www.cdc.gov/nchs/nhanes/, accessed on 6th, March 2023).
