# Supplementary material for: Association of obstructive sleep apnea symptoms with all‐cause mortality and cause‐specific mortality in adults with or without diabetes: A cohort study based on the NHANES
Source: J Diabetes. 2024 Apr 10;16(4):e13538. doi: 10.1111/1753-0407.13538 (PMC11006614; doi:10.1111/1753-0407.13538)
Supplement: Supplementary file 1 — Data S1. Supplementary Information. [file JDB-16-e13538-s001.docx]

All-cause mortality

Survival

Time, year

Cardiovascular mortality

Survival

Time, year

Cancer mortality

Survival

Time, year

Cardiovascular mortality

Survival

Time, year

All-cause mortality

Survival

Time, year

Cancer mortality

Survival

Time, year

A

B

Log-rank P<0.0001

Log-rank P<0.0001

Log-rank P<0.01

Log-rank P<0.0001

Log-rank P<0.0001

Log-rank P<0.001

Q1

Q2

Q3

Q4

**Supplemental Figure 1.** Kaplan-Meier survival curves (crude) were performed to analyze the difference of mortality among the OSAS.MAP10 quartiles groups (Q1-Q4). A, participants without diabetes. B, patients with pre-diabetes.

**Supplementary Table 1**. Demographic characteristics of US adults with or without OSAS.

| Characteristic | Non-OSAS，N = 12424 (68%) ^†^ | OSAS, N = 5718 (32%) ^†^ | P Value^‡^ |
| --- | --- | --- | --- |
| Age, years | 45.8 (0.3) | 48.5 (0.4) | **<0.001** |
| Sex |  |  | **<0.001** |
| Female | 6612 (54.1%) | 2626 (45.3%) |  |
| Male | 5812 (45.9%) | 3092 (54.7%) |  |
| Race/Ethnicity |  |  | **0.028** |
| Hispanic | 1185 (5.7%) | 567 (5.2%) |  |
| Non-Hispanic black | 2687 (11.1%) | 1238 (11.0%) |  |
| Non- Hispanic white | 4963 (66.2%) | 2429 (68.5%) |  |
| Other | 3589 (17.0%) | 1484 (15.2%) |  |
| BMI, kg/m^2^ |  |  | **<0.001** |
| <25 | 4149 (35.6%) | 1020 (18.1%) |  |
| 25-30 | 4261 (33.3%) | 1783 (30.8%) |  |
| ≥30 | 4014 (31.1%) | 2915 (51.1%) |  |
| Education |  |  | **0.002** |
| College and above | 6487 (61.6%) | 2928 (58.3%) |  |
| High school or equivalent | 2863 (23.4%) | 1393 (26.4%) |  |
| Less than high school | 3066 (14.9%) | 1393 (15.3%) |  |
| Family income poverty ratio |  |  | 0.3 |
| ≤1 | 2219 (12.7%) | 1018 (12.7%) |  |
| 1-3 | 4782 (35.1%) | 2296 (36.7%) |  |
| >3 | 4303 (52.1%) | 1920 (50.6%) |  |
| Smoking status |  |  | **<0.001** |
| Former | 2808 (23.5%) | 1515 (26.6%) |  |
| Never | 7341 (58.3%) | 2818 (49.3%) |  |
| Now | 2269 (18.2%) | 1380 (24.2%) |  |
| Drinking status |  |  | **<0.001** |
| Never | 1760 (12.2%) | 558 (8.0%) |  |
| Former | 1561 (10.9%) | 796 (13.3%) |  |
| Mild | 3603 (37.1%) | 1789 (38.5%) |  |
| Moderate | 1758 (18.7%) | 799 (17.4%) |  |
| Heavy | 2139 (21.1%) | 1061 (22.8%) |  |
| HbA1c, % | 5.5 (0.0) | 5.7 (0.0) | **<0.001** |
| Diabetes Status |  |  | **<0.001** |
| Non-diabetes | 7080 (62.3%) | 2561 (49.7%) |  |
| Prediabetes | 3405 (26.1%) | 1813 (32.6%) |  |
| Diabetes Status | 1939 (11.7%) | 1344 (17.7%) |  |
| COPD | 433 (3.1%) | 341 (5.1%) | **<0.001** |
| Asthma | 1537 (13.3%) | 987 (17.1%) | **<0.001** |
| Chronic kidney disease | 2065 (13.5%) | 1096 (15.8%) | **0.002** |
| Hypertension | 4615 (32.2%) | 2852 (45.8%) | **<0.001** |
| Hyperlipidemia | 8095 (64.0%) | 4215 (73.6%) | **<0.001** |
| Cardiovascular disease | 1153 (7.0%) | 792 (10.6%) | **<0.001** |
| Diabetes medication use |  |  | **<0.001** |
| None | 5925 (47.1%) | 2043 (36.2%) |  |
| Other | 5285 (45.5%) | 2780 (52.4%) |  |
| Oral medication or insulin | 1207 (7.4%) | 890 (11.4%) |  |

† Mean (standard deviation) for continuous variable; n (%) for categorical variable.

‡ Wilcoxon rank-sum test for complex survey samples; chi-squared test with Rao & Scott's second-order correction.

BMI, body mass index; HbA1c, hemoglobin A1c; COPD, chronic obstructive pulmonary disease; OSAS, obstructive sleep apnea symptoms.

**Supplementary Table 2.** Demographic characteristics of US adults with diabetes according to quartiles of OSAS.MAP10.

|  | | | OSAS.MAP10 | | | |  |
| --- | --- | --- | --- | --- | --- | --- | --- |
| Characteristic | N | Overall, N = 3283 (100%) | Q1, N = 219 (7.0%)^†^ | Q2, N = 582 (17%)^†^ | Q3, N = 944 (28%)^†^ | Q4, N = 1538 (48%)^†^ | P Value^‡^ |
| Age, years | 3283 | 58.9 (0.4) | 49.9 (1.3) | 57.1 (0.8) | 58.7 (0.7) | 60.9 (0.5) | **<0.001** |
| Sex | 3283 |  |  |  |  |  | **<0.001** |
| Female |  | 1543 (49%) | 199 (89%) | 437 (77%) | 526 (57%) | 381 (28%) |  |
| Male |  | 1740 (51%) | 20 (11%) | 145 (23%) | 418 (43%) | 1157 (72%) |  |
| Race/ethnicity | 3283 |  |  |  |  |  | **<0.001** |
| Hispanic |  | 350 (5.8%) | 22 (6.6%) | 63 (6.5%) | 118 (6.9%) | 147 (4.7%) |  |
| Non-Hispanic black |  | 835 (14%) | 50 (14%) | 150 (17%) | 259 (16%) | 376 (12%) |  |
| Non- Hispanic white |  | 1138 (62%) | 56 (50%) | 171 (54%) | 297 (61%) | 614 (68%) |  |
| Other |  | 960 (18%) | 91 (29%) | 198 (23%) | 270 (16%) | 401 (15%) |  |
| BMI, kg/m^2^ | 3283 |  |  |  |  |  | **<0.001** |
| <25 |  | 469 (12%) | 136 (58%) | 167 (27%) | 103 (7.9%) | 63 (3.0%) |  |
| 25-30 |  | 953 (26%) | 66 (32%) | 249 (43%) | 328 (31%) | 310 (17%) |  |
| ≥30 |  | 1861 (61%) | 17 (10%) | 166 (31%) | 513 (61%) | 1,165 (80%) |  |
| Education | 3280 |  |  |  |  |  | 0.092 |
| College and above |  | 1408 (52%) | 102 (57%) | 221 (47%) | 423 (55%) | 662 (52%) |  |
| High school or equivalent |  | 783 (27%) | 42 (20%) | 158 (28%) | 206 (23%) | 377 (29%) |  |
| Less than high school |  | 1089 (21%) | 74 (22%) | 202 (25%) | 315 (21%) | 498 (19%) |  |
| Family income poverty ratio | 2950 |  |  |  |  |  | **0.004** |
| ≤1 |  | 652 (15%) | 42 (16%) | 141 (20%) | 189 (15%) | 280 (13%) |  |
| 1-3 |  | 1372 (41%) | 96 (45%) | 246 (44%) | 396 (43%) | 634 (38%) |  |
| >3 |  | 926 (44%) | 57 (38%) | 135 (37%) | 263 (42%) | 471 (49%) |  |
| Smoking status | 3283 |  |  |  |  |  | **<0.001** |
| Former |  | 1075 (33%) | 29 (16%) | 130 (26%) | 283 (32%) | 633 (39%) |  |
| Never |  | 1689 (51%) | 149 (60%) | 346 (56%) | 494 (50%) | 700 (49%) |  |
| Now |  | 519 (16%) | 41 (24%) | 106 (18%) | 167 (17%) | 205 (12%) |  |
| Drinking status | 2803 |  |  |  |  |  | **<0.001** |
| Never |  | 517 (16%) | 57 (28%) | 134 (23%) | 163 (18%) | 163 (11%) |  |
| Former |  | 665 (19%) | 31 (20%) | 99 (18%) | 175 (15%) | 360 (22%) |  |
| Mild |  | 929 (38%) | 44 (21%) | 120 (27%) | 257 (36%) | 508 (44%) |  |
| Moderate |  | 328 (14%) | 22 (15%) | 64 (16%) | 115 (16%) | 127 (13%) |  |
| Heavy |  | 364 (13%) | 23 (15%) | 74 (16%) | 104 (15%) | 163 (10%) |  |
| OSAS.MAP10 | 3283 | 5.68 (0.05) | 1.08 (0.04) | 2.82 (0.03) | 5.08 (0.03) | 7.74 (0.03) | **<0.001** |
| COPD | 3283 | 241 (6.7%) | 12 (9.2%) | 27 (4.3%) | 56 (6.1%) | 146 (7.7%) | 0.13 |
| Asthma | 3283 | 517 (16%) | 28 (17%) | 82 (17%) | 140 (15%) | 267 (16%) | 0.7 |
| Chronic kidney disease | 3148 | 1272 (36%) | 68 (31%) | 211 (30%) | 341 (33%) | 652 (40%) | **0.010** |
| Hypertension | 3282 | 2330 (69%) | 115 (48%) | 377 (62%) | 665 (70%) | 1173 (75%) | **<0.001** |
| Hyperlipidemia | 3283 | 2809 (87%) | 157 (72%) | 503 (88%) | 817 (87%) | 1332 (90%) | **<0.001** |
| Cardiovascular disease | 3283 | 826 (23%) | 24 (11%) | 103 (17%) | 222 (20%) | 477 (30%) | **<0.001** |
| Diabetes medication use | 3281 |  |  |  |  |  | **<0.001** |
| None |  | 407 (11%) | 46 (20%) | 80 (13%) | 124 (11%) | 157 (8.8%) |  |
| Other |  | 790 (26%) | 49 (25%) | 159 (32%) | 224 (25%) | 358 (24%) |  |
| Oral medication or insulin |  | 2084 (63%) | 124 (55%) | 343 (55%) | 594 (63%) | 1023 (67%) |  |

^†^ Mean (standard deviation) for continuous variable; n (%) for categorical variable.

^‡^ Wilcoxon rank-sum test for complex survey samples; chi-squared test with Rao & Scott's second-order correction.

BMI, body mass index; COPD, chronic obstructive pulmonary disease; OSAS, obstructive sleep apnea symptoms; MAP, multivariable apnea prediction; OSAS.MAP10, obstructive sleep apnea symptoms multivariable apnea prediction *10.

**Supplementary Table 3.** Weighted association between OSAS.MAP10 with all-cause mortality and cause-specific mortality in no diabetes.

|  |  | Male | | | Female | | |
| --- | --- | --- | --- | --- | --- | --- | --- |
| Group | Characteristic | HR | 95% CI | P-value | HR | 95% CI | P-value |
| All-cause mortality | Model 1 | 1.27 | 1.16, 1.39 | **<0.001** | 1.29 | 1.20, 1.37 | **<0.001** |
|  | Model 2 | 1.25 | 1.14, 1.37 | **<0.001** | 1.21 | 1.12, 1.30 | **<0.001** |
|  | Model 3 | 1.12 | 1.03, 1.22 | **0.008** | 1.05 | 0.97, 1.13 | 0.2 |
| Cardiovascular mortality | Model 1 | 1.46 | 1.23, 1.74 | **<0.001** | 1.25 | 1.04, 1.51 | **0.016** |
|  | Model 2 | 1.28 | 1.22, 1.35 | **<0.001** | 1.2 | 1.01, 1.43 | **0.035** |
|  | Model 3 | 1.13 | 1.05, 1.20 | **<0.001** | 0.86 | 0.63, 1.16 | 0.3 |
| Cancer mortality | Model 1 | 1.26 | 1.06, 1.51 | **0.009** | 1.38 | 1.23, 1.55 | **<0.001** |
|  | Model 2 | 1.24 | 1.05, 1.45 | **0.009** | 1.28 | 1.13, 1.44 | **<0.001** |
|  | Model 3 | 1.09 | 0.90, 1.31 | 0.4 | 1.1 | 0.96, 1.26 | 0.2 |

Model 1: adjusted for age, sex, race/ethnicity, and BMI.

Model 2: adjusted for all variables in model 1 and other risk factors for death, including education, smoking status, drinking status, and family income poverty ratio.

Model 3: adjusted for all variables in model 2 and other risk factors for death, including cardiovascular disease, hypertension, hypercholesterolemia, chronic obstructive pulmonary disease, asthma, and chronic kidney disease.

HR, hazard ratios; 95%CI, 95% confidence interval; OSAS.MAP10, obstructive sleep apnea symptoms multivariable apnea prediction *10.

**Supplementary Table 4.** Weighted association between OSAS.MAP10 with all-cause mortality and cause-specific mortality in prediabetes.

|  |  | Male | | | Female | | |
| --- | --- | --- | --- | --- | --- | --- | --- |
| Group | Characteristic | HR | 95% CI | P-value | HR | 95% CI | P-value |
| All-cause mortality | Model 1 | 1.13 | 1.03, 1.23 | **0.007** | 1.07 | 0.98, 1.16 | 0.12 |
|  | Model 2 | 1.11 | 1.02, 1.20 | **0.021** | 1.03 | 0.94, 1.14 | 0.5 |
|  | Model 3 | 1.01 | 0.93, 1.09 | 0.9 | 0.99 | 0.89, 1.10 | 0.9 |
| Cardiovascular mortality | Model 1 | 1.17 | 1.05, 1.31 | **0.006** | 1.24 | 1.02, 1.50 | **0.034** |
|  | Model 2 | 1.16 | 1.04, 1.30 | **0.01** | 1.14 | 0.93, 1.39 | 0.2 |
|  | Model 3 | 1.02 | 0.92, 1.14 | 0.7 | 1.12 | 0.87, 1.44 | 0.4 |
| Cancer mortality | Model 1 | 1.12 | 0.97, 1.30 | 0.13 | 1.03 | 0.88, 1.21 | 0.7 |
|  | Model 2 | 1.09 | 0.94, 1.27 | 0.2 | 1.03 | 0.88, 1.21 | 0.7 |
|  | Model 3 | 0.98 | 0.78, 1.21 | 0.8 | 1.02 | 0.85, 1.23 | 0.8 |

Model 1: adjusted for age, sex, race/ethnicity, and BMI.

Model 2: adjusted for all variables in model 1 and other risk factors for death, including education, smoking status, drinking status, and family income poverty ratio.

Model 3: adjusted for all variables in model 2 and other risk factors for death, including cardiovascular disease, hypertension, hypercholesterolemia, chronic obstructive pulmonary disease, asthma, and chronic kidney disease.

HR, hazard ratios; 95%CI, 95% confidence interval; OSAS.MAP10, obstructive sleep apnea symptoms multivariable apnea prediction *10.

**Supplementary Table 5.** Weighted association between OSAS.MAP10 with all-cause mortality and cause-specific mortality in diabetes.

|  |  | Male | | | Female | | |
| --- | --- | --- | --- | --- | --- | --- | --- |
| Group | Characteristic | HR | 95% CI | P-value | HR | 95% CI | P-value |
| All-cause mortality | Model 1 | 1.13 | 1.03, 1.25 | **0.011** | **0.94** | **0.87, 1.03** | **0.2** |
|  | Model 2 | 1.12 | 1.00, 1.24 | **0.041** | **0.93** | **0.85, 1.01** | **0.08** |
|  | Model 3a | 1.08 | 0.97, 1.21 | 0.14 | **0.88** | **0.80, 0.97** | **0.013** |
| Cardiovascular mortality | Model 1 | 1.1 | 0.93, 1.29 | 0.3 | 0.98 | 0.86, 1.11 | 0.7 |
|  | Model 2 | 1.07 | 0.90, 1.26 | 0.4 | 0.95 | 0.84, 1.09 | 0.5 |
|  | Model 3a | 1.02 | 0.87, 1.18 | 0.8 | 0.88 | 0.74, 1.04 | 0.14 |
| Cancer mortality | Model 1 | 1.26 | 1.00, 1.59 | 0.048 | 0.98 | 0.86, 1.11 | 0.7 |
|  | Model 2 | 1.21 | 0.98, 1.49 | 0.076 | 0.85 | 0.63, 1.15 | 0.3 |
|  | Model 3a | 1.16 | 0.93, 1.46 | 0.2 | 0.86 | 0.65, 1.12 | 0.3 |

Model 1: adjusted for age, sex, race/ethnicity, and BMI.

Model 2: adjusted for all variables in model 1 and other risk factors for death, including education, smoking status, drinking status, and family income poverty ratio.

Model 3a: adjusted for all variables in model 2 and other risk factors for death, including cardiovascular disease, hypertension, hypercholesterolemia, chronic obstructive pulmonary disease, asthma, chronic kidney disease, HbA1c, and diabetes medication usage.

HR, hazard ratios; 95%CI, 95% confidence interval; OSAS.MAP10, obstructive sleep apnea symptoms multivariable apnea prediction *10.

**Supplementary Table 6.** Stratified analyses with two-piecewise COX regression exploring the relationship between OSAS.MAP10 and all-cause mortality in diabetes.

| Subgroups | HR (95% CI)^1^ | P for trend | P for interaction |
| --- | --- | --- | --- |
| Age, years |  |  | 0.056 |
| ≤60 | 1.067 (0.924, 1.233) | 0.376 |  |
| >60 | 1.010 (0.935, 1.091) | 0.799 |  |
| Sex |  |  | **0.006** |
| Male | 1.121 (1.000, 1.256) | **0.050** |  |
| Female | 0.979 (0.871, 1.101) | 0.727 |  |
| BMI, kg/m^2^ |  |  | **0.036** |
| <25 | 0.967 (0.807, 1.158) | 0.714 |  |
| 25-30 | 0.957 (0.813, 1.127) | 0.599 |  |
| ≥30 | 1.110 (0.965, 1.276) | 0.144 |  |
| Race/ethnicity |  |  | 0.185 |
| Non-White | 0.974 (0.874, 1.086) | 0.639 |  |
| Non-Black | 1.208 (1.075, 1.358) | 0.002 |  |
| Hispanic | 1.098 (0.821, 1.469) | 0.529 |  |
| Other | 1.010 (0.826, 1.235) | 0.923 |  |
| Education |  |  | 0.096 |
| College and above | 0.977 (0.845, 1.131) | 0.757 |  |
| High school or equivalent | 1.014 (0.894, 1.150) | 0.829 |  |
| Less than high school | 1.140 (1.031, 1.261) | **0.010** |  |

^1^hazard ratios (95% confidence interval).

OSAS.MAP10, obstructive sleep apnea symptoms multivariable apnea prediction *10.

**Supplementary Table 7.** Association of OSAS.MAP10 with all-cause mortality in diabetic participants, after excluding participants who died within two years (n=2338).

|  | | OSAS.MAP10<6.65 | | | OSAS.MAP10 ≥6.65 | | |
| --- | --- | --- | --- | --- | --- | --- | --- |
| Group | Characteristic | HR | 95% CI | P-value | HR | 95% CI | P-value |
| All-cause mortality | Model 1 | 0.94 | 0.83, 1.06 | 0.3 | 1.38 | 1.08, 1.77 | **0.010** |
|  | Model 2 | 0.94 | 0.84, 1.06 | 0.3 | 1.35 | 1.02, 1.78 | **0.036** |
|  | Model 3a | 0.94 | 0.84, 1.05 | 0.3 | 1.42 | 1.10, 1.84 | **0.007** |

Model 1: adjusted for age, sex, race/ ethnicity, and BMI.

Model 2: adjusted for all variables in model 1 and other risk factors for death, including education, smoking status, drinking status, and Family income poverty ratio.

Model 3a: adjusted for all variables in model 2 and other risk factors for death, including cardiovascular disease, hypertension, hypercholesterolemia, chronic obstructive pulmonary disease, asthma, chronic kidney disease, HbA1c, and diabetes medication usage.

HR, hazard ratios; 95%CI, 95% confidence interval.

OSAS.MAP10, obstructive sleep apnea symptoms multivariable apnea prediction *10.

**Supplementary Table 8.** Association of OSAS.MAP10 with all-cause mortality in diabetic participants, after excluding participants with history of CVD (n= 1841).

|  | | OSAS.MAP10<6.65 | | | OSAS.MAP10 ≥6.65 | | |
| --- | --- | --- | --- | --- | --- | --- | --- |
| Group | Characteristic | HR | 95% CI | P-value | HR | 95% CI | P-value |
| All-cause mortality | Model 1 | 0.90 | 0.78, 1.03 | 0.12 | 1.48 | 1.05, 2.08 | **0.024** |
|  | Model 2 | 0.90 | 0.79, 1.03 | 0.13 | 1.50 | 1.02, 2.19 | **0.039** |
|  | Model 3a | 0.91 | 0.80, 1.04 | 0.2 | 1.80 | 1.21, 2.67 | **0.004** |

Model 1: adjusted for age, sex, race/ethnicity, and BMI.

Model 2: adjusted for all variables in model 1 and other risk factors for death, including education, smoking status, drinking status, and family income poverty ratio.

Model 3a: adjusted for all variables in model 2 and other risk factors for death, including hypertension, hypercholesterolemia, chronic obstructive pulmonary disease, asthma, chronic kidney disease, HbA1c, and diabetes medication usage (participants with CVDs have been excluded).

CVD, cardiovascular disease; HR, hazard ratios; 95%CI, 95% confidence intervals; OSAS.MAP10, obstructive sleep apnea symptoms multivariable apnea prediction *10.

**Supplementary Table 9.** Association of OSAS.MAP10 with all-cause mortality in diabetic participants, after excluding participants using the diabetes medication (n=917).

|  | | OSAS.MAP10<6.65 | | | OSAS.MAP10 ≥6.65 | | |
| --- | --- | --- | --- | --- | --- | --- | --- |
| Group | Characteristic | HR | 95% CI | P-value | HR | 95% CI | P-value |
| All-cause mortality | Model 1 | 0.88 | 0.75, 1.03 | 0.10 | 1.05 | 0.77, 1.43 | 0.8 |
|  | Model 2 | 0.90 | 0.78, 1.04 | 0.2 | 1.09 | 0.74, 1.59 | 0.7 |
|  | Model 3a | 0.86 | 0.74, 1.00 | 0.051 | 1.11 | 0.74, 1.67 | 0.6 |

Model 1: adjusted for age, sex, race/ethnicity, and BMI.

Model 2: adjusted for all variables in model 1 and other risk factors for death, including education, smoking status, drinking status, and family income poverty ratio.

Model 3a: adjusted for all variables in model 2 and other risk factors for death, including cardiovascular disease, hypertension, hypercholesterolemia, chronic obstructive pulmonary disease, asthma, chronic kidney disease, and HbA1c (participants using the diabetes medicines have been excluded).

HR, hazard ratios; 95%CI, 95% confidence intervals; OSAS.MAP10, obstructive sleep apnea symptoms multivariable apnea prediction *10.

**Supplementary Table 10.** Association of OSAS.MAP10 with cancer mortality in diabetic participants, after excluding participants with history of CVD (n= 1636).

|  | | OSAS.MAP10<6.51 | | | OSAS.MAP10 ≥6.51 | | |
| --- | --- | --- | --- | --- | --- | --- | --- |
| Group | Characteristic | HR | 95% CI | P-value | HR | 95% CI | P-value |
| Cancer mortality | Model 1 | 0.91 | 0.68, 1.23 | 0.5 | 2.17 | 1.15, 4.09 | **0.017** |
|  | Model 2 | 0.94 | 0.67, 1.31 | 0.7 | 2.11 | 1.12, 3.97 | **0.021** |
|  | Model 3a | 0.99 | 0.76, 1.29 | >0.9 | 3.39 | 1.16, 9.90 | **0.025** |

Model 1: adjusted for age, sex, race/ethnicity, and BMI.

Model 2: adjusted for all variables in model 1 and other risk factors for death, including education, smoking status, drinking status, and family income poverty ratio.

Model 3a: adjusted for all variables in model 2 and other risk factors for death, including hypertension, hypercholesterolemia, chronic obstructive pulmonary disease, asthma, chronic kidney disease, HbA1c, and diabetes medication usage (participants with CVDs have been excluded).

CVD, cardiovascular disease; HR, hazard ratios; 95%CI, 95% confidence intervals; OSAS.MAP10, obstructive sleep apnea symptoms multivariable apnea prediction *10.

**Supplementary Table 11.** Association of OSAS.MAP10 with cancer mortality in diabetic participants, after excluding participants using the diabetes medication (n=785).

|  | | OSAS.MAP10<6.51 | | | OSAS.MAP10≥6.51 | | |
| --- | --- | --- | --- | --- | --- | --- | --- |
| Group | Characteristic | HR | 95% CI | P-value | HR | 95% CI | P-value |
| Cancer mortality | Model 1 | 1.04 | 0.75, 1.46 | 0.8 | 1.81 | 1.04, 3.16 | **0.036** |
|  | Model 2 | 1.11 | 0.75, 1.65 | 0.6 | 2.05 | 0.88, 4.76 | 0.10 |
|  | Model 3a | 1.04 | 0.82, 1.32 | 0.7 | 2.41 | 1.21, 4.81 | **0.012** |

Model 1: adjusted for age, sex, race/ethnicity, and BMI.

Model 2: adjusted for all variables in model 1 and other risk factors for death, including education, smoking status, drinking status, and family income poverty ratio.

Model 3a: adjusted for all variables in model 2 and other risk factors for death, including cardiovascular disease, hypertension, hypercholesterolemia, Chronic Obstructive Pulmonary Disease, Asthma, chronic kidney disease, and HbA1c (participants using the diabetes medicines have been excluded).

HR, hazard ratios; 95%CI, 95% confidence intervals; OSAS.MAP10, obstructive sleep apnea symptoms multivariable apnea prediction *10.

**Supplementary Table 12.** Association of OSAS.MAP10 with cancer mortality in diabetic participants, after excluding participants who died within two years (n=2020).

|  | | OSAS.MAP10<6.51 | | | OSAS.MAP10 ≥6.51 | | |
| --- | --- | --- | --- | --- | --- | --- | --- |
| Group | Characteristic | HR | 95% CI | P-value | HR | 95% CI | P-value |
| Cancer mortality | Model 1 | 1.02 | 0.75, 1.40 | 0.9 | 1.75 | 1.09, 2.82 | **0.020** |
|  | Model 2 | 1.06 | 0.76, 1.47 | 0.7 | 1.58 | 0.93, 2.71 | 0.092 |
|  | Model 3a | 1.09 | 0.86, 1.39 | 0.5 | 1.62 | 0.99, 2.66 | 0.055 |

Model 1: adjusted for age, sex, race/ethnicity, and BMI.

Model 2: adjusted for all variables in model 1 and other risk factors for death, including education, smoking status, drinking status, and family income poverty ratio.

Model 3a: adjusted for all variables in model 2 and other risk factors for death, including cardiovascular disease, hypertension, hypercholesterolemia, chronic obstructive pulmonary disease, asthma, chronic kidney disease, HbA1c, and diabetes medication usage.

HR, hazard ratios; 95%CI, 95% confidence intervals; OSAS.MAP10, obstructive sleep apnea symptoms multivariable apnea prediction *10.
